# Amino acid profile alteration in age-related atrial fibrillation

**DOI:** 10.1186/s12967-024-05028-7

**Published:** 2024-03-09

**Authors:** Yunying Huang, Qiuzhen Lin, Yong Zhou, Jiayi Zhu, Yingxu Ma, Keke Wu, Zuodong Ning, Zixi Zhang, Na Liu, Mohan Li, Yaozhong Liu, Tao Tu, Qiming Liu

**Affiliations:** 1 Department of Cardiovascular Medicine, The Second Xiangya Hospital, Central South University, No.139 Middle Renmin Road, Changsha, 410011 Hunan People’s Republic of China; 2 Modern Cardiovascular Disease Clinical Technology Research Center of Hunan Province, Changsha, 410011 Hunan People’s Republic of China; 3 Cardiovascular Disease Research Center of Hunan Province, Changsha, 410011 Hunan People’s Republic of China; 4 Research Institute of Blood Lipid and Atherosclerosis, Central South University, Changsha, 410011 Hunan People’s Republic of China; 5 Department of Geriatrics, Peking Union Medical College Hospital, Chinese Academy of Medical Sciences, Beijing, 100730 People’s Republic of China; 6 Department of Internal Medicine, Frankel Cardiovascular Center, University of Michigan, Ann Arbor, MI USA

**Keywords:** Atrial fibrillation, Aging, Age-related atrial fibrillation, Amino acids, Metabolomics, Gut microbiota

## Abstract

**Background:**

Amino acids (AAs) are one of the primary metabolic substrates for cardiac work. The correlation between AAs and both atrial fibrillation (AF) and aging has been documented. However, the relationship between AAs and age-related AF remains unclear.

**Methods:**

Initially, the plasma AA levels of persistent AF patients and control subjects were assessed, and the correlations between AA levels, age, and other clinical indicators were explored. Subsequently, the age-related AF mouse model was constructed and the untargeted myocardial metabolomics was conducted to detect the level of AAs and related metabolites. Additionally, the gut microbiota composition associated with age-related AF was detected by a 16S rDNA amplicon sequencing analysis on mouse fecal samples.

**Results:**

Higher circulation levels of lysine (Student’s t-test, *P* = 0.001), tyrosine (*P* = 0.002), glutamic acid (*P* = 0.008), methionine (*P* = 0.008), and isoleucine (*P* = 0.014), while a lower level of glycine (*P* = 0.003) were observed in persistent AF patients. The feature AAs identified by machine learning algorithms were glutamic acid and methionine. The association between AAs and age differs between AF and control subjects. Distinct patterns of AA metabolic profiles were observed in the myocardial metabolites of aged AF mice. Aged AF mice had lower levels of Betaine, L-histidine, L-alanine, L-arginine, L-Pyroglutamic acid, and L-Citrulline compared with adult AF mice. Aged AF mice also presented a different gut microbiota pattern, and its functional prediction analysis showed AA metabolism alteration.

**Conclusion:**

This study provided a comprehensive network of AA disturbances in age-related AF from multiple dimensions, including plasma, myocardium, and gut microbiota. Disturbances of AAs may serve as AF biomarkers, and restoring their homeostasis may have potential benefits for the management of age-related AF.

**Supplementary Information:**

The online version contains supplementary material available at 10.1186/s12967-024-05028-7.

## Introduction

Atrial fibrillation (AF) is the most common sustained cardiac arrhythmia in clinics with significant morbidity and mortality. The estimated prevalence of AF in adults ranges between 2 and 4% [1], and its rise is projected due to the extended longevity [2]. Advanced age is the most prominent risk factor for the incidence, prevalence [2, 3], and progression [4–7] of AF. Participants aged 80–89 years had a 9.33-fold increased risk of AF compared to those aged 50–59 years [8]. It remains uncertain whether aging in age-related AF is a determinant of adverse prognosis or rather a marker of an underlying progressive substrate. Thus, an in-depth understanding of its mechanism is still expected.

While electrical, structural, and contractile remodeling are well-known contributors to AF incidence and progression [9], the role of cardiac metabolic remodeling in AF remains largely unexplored. Atrial fibrillation is characterized by irregular high-frequency excitation and contraction, which disrupts the balance between metabolic demand and supply and causes metabolic stress [10, 11]. Recent metabolomic, proteomic, and transcriptomic studies suggested the involvement of metabolism in AF pathophysiology [12–14].

Amino acids (AAs) are one of the primary metabolic substrates for cardiac work, and their homeostasis affects cardiovascular physiology and pathology [15, 16]. Atrial appendage tissues from persistent AF patients had higher levels of ketogenic AAs and glycine [12]. Plasma AA profiles of AF patients had lower circulating level of 4-hydroxy-pyrrolidine-2-carboxylic acid (4HP2C) [17], implying defective proline metabolism. The correlation between disturbed AAs and aging has been reported, and the majority of the report revealed decreased branched-chain AAs (BCAAs) and increased citrulline with old age [18–22]. Proposed mechanisms included aging-related reduced dietary protein intake, reduced muscle mass, and altered gluconeogenesis and urea cycle metabolism [23, 24].

The observed AAs profile disturbances in both AF and aging suggest that AAs may participate in the development of age-related AF. However, AA alterations in age-related AF have not been investigated. To address this gap, our study analyzed plasma AA levels of persistent AF patients and their relationship with age, myocardial metabolites of aged AF mouse models, and gut microbiota of aged AF mouse models. Our study illustrated that AA disturbance may be one of the potential mechanisms of metabolic remodeling in age-related AF.

## Material and method

### Patient enrollment

Patients admitted to the cardiology departments of the Second Xiangya Hospital of Central South University between September 2021 and January 2022 were selected. Eligible patients were diagnosed with persistent AF and with ages between 18 and 80 years old. According to the 2020 ESC guideline, persistent AF is defined as AF consistently sustained for more than 7 days, including episodes terminated by cardioversion after the 7-day threshold [1]. The exclusion criteria were: (1) severe non-cardiac diseases, (2) expected life expectancy of less than 1 year, and (3) other health behaviors that might affect the study, such as alcoholism. This study was approved by the Ethics Committee of Second Xiangya Hospital of Central South University. All procedures performed in this study involving human participants were following the ethical standards of the institutional and/or national research committee and with the 1964 Helsinki Declaration and its later amendments or comparable ethical standards. Informed consent was gained from all subjects.

### Demographic and biochemical measurements

The hospital medical records were accessed to retrieve the participants' demographic information and medical history. Peripheral blood samples were collected from the participants for biochemical measurements, including blood routine tests, liver function, renal function, thyroid function, blood lipid, blood glucose, creatine kinase, etc. The cardiac ultrasound parameters were also recorded.

### Patient plasma collection and AAs detection

Plasma samples were collected from 30 persistent AF patients and 30 corresponding control participants. After an overnight fasting, peripheral blood samples were obtained using 5 mL vacutainer tubes containing the anticoagulant agent ethylenediaminetetraacetic acid. These samples were then centrifuged at 2500 rpm for 15 min (min) at 4 °C. The resulting plasma samples were stored at –80 °C until further analysis.

Appropriate amounts of plasma were taken and mixed with methanol at a ratio of 1:5 (v/v). The mixture was subjected to ultrasonication for 20 min, followed by centrifugation (4 °C, 12,000 rpm, 20 min) to collect the supernatant. A 1:1 ratio was used to mix the supernatant with the internal standard, 4-aminobenzoic acid. The mixture was vortexed and then centrifuged at 12,000 rpm for 10 min. The resulting supernatant was used for AA detection by the liquid chromatography-mass spectrometry (LC–MS) assay (QTRAP 6500 + System, SCIEX).

The significantly different AAs were shown in the volcano plot. To assess their diagnostic efficacy, receiver operating characteristic (ROC) curves were plotted and the area under the curve (AUC) was calculated. The automated identification of important AAs was conducted by multivariate machine learning algorithms, including LASSO (least absolute shrinkage and selection operator), SVM-RFE (Support Vector Machine-Recursive Feature Elimination), Random Forests, and XGBoost (Extreme Gradient Boosting). In LASSO, the optimal regression model and variables were obtained using lambda.1se. SVM-RFE and Random Forests selected crucial AAs based on the minimum cross-validation error. XGBoost determined the optimal number of iterations with the lowest logarithmic loss during cross-validation, resulting in a model. Top feature importance AAs were selected in the XGBoost model. The integration of outcomes from these four machine learning algorithms identified feature AAs strongly correlated with AF with high accuracy. Correlation analysis was conducted to examine the correlations between AAs and clinical characteristics of participants.

### Atrial fibrillation mouse model establishment

C57BL/6 J adult male mice (8–12 weeks old) and C57BL/6 J aged male mice (13 months old) were obtained from SJA Laboratory Animal Co. Ltd (Changsha, China). The mice were housed under controlled temperature and lighting, with ad libitum access to water and a standard chow diet. The mice were randomly assigned to four groups: adult control (Group A, Aadcon), adult AF (Group B, BadAF), aged control (Group C, Cagcon), and aged AF (Group D, DagAF). Mice of the AF groups (Group B and Group D) received daily injections of a mixture solution of acetylcholine (Ach, 66 μg/kg body weight; Shanghai Macklin Biochemical Ltd., Shanghai, China) and calcium chloride (CaCl_2_, 10 mg/kg body weight; Shanghai Macklin Biochemical Ltd., Shanghai, China) by medial canthal vein (i.v.) for 4 weeks, while the mice of the control groups (Group A and Group C) were injected with saline [25–27].

To confirm the establishment of the AF mouse models, surface electrocardiogram (ECG, Rhythmia Mapping System, Shanghai Hongtong Industrial Co., Ltd) and echocardiography (VINNO Technology Suzho Co., Ltd.) were recorded. The cardiac structural and functional parameters were recorded, including left atrial area (LAA), left ventricular internal diameter at end-diastole (LVIDs), left ventricular internal diameter at end-systole (LVIDd), fractional shortening (FS), and left ventricular ejection fraction (LVEF).

Myocardial histological changes in the mouse model were detected. Following overnight fixation in 4% paraformaldehyde, the mice's heart tissues were paraffin-embedded and sectioned into 5-μm-thick slices. Myocardial fibrosis was assessed through Masson-stained sections. Five optical fields were then examined per section (Image J), with the evaluation conducted in a blinded manner.

The animal use protocol listed above has been reviewed and approved by the Institutional Animal Care and Use Committee (IACUC), The Second Xiangya Hospital, Central South University, China (Approval number: 2022730).

### Myocardial untargeted metabolomics of AF mouse model

Heart tissues of the mouse model were collected and used for myocardial untargeted metabolomics analysis. Mice heart tissues were homogenized with 200 µL of water. Then, 800 µL of methanol/acetonitrile (1:1, v/v) was added to the homogenized solution. The mixtures were then centrifuged (4 °C, 14000 × *g*, 15 min) and the supernatants were collected and dried. The dried supernatants were redissolved in 100 µL of acetonitrile/water (1:1, v/v) solvent for LC–MS/MS analysis. The LC–MS/MS analysis was performed by UHPLC (1290 Infinity LC, Agilent Technologies) and quadrupole time-of-flight (AB Sciex TripleTOF 6600).

The raw data were converted and imported into XCMS software for data analysis. Analysis was performed for all metabolites detected in positive and negative ion modes, including those not identified. The univariate analysis included fold change (FC) analysis and Student's t-test. Differential metabolites in univariate analysis were defined as those with FC > 1.5 or FC < 0.67 and Student's t-test *P*-value < 0.05 and shown in the volcano plot. To better reveal features of these complex datasets, multi-dimensional statistical analyses were used [28], including principal component analysis (PCA) [29] and orthogonal partial least-squares discriminant analysis (OPLS-DA) [30, 31]. The robustness of each analysis model was evaluated by sevenfold cross-validation and response permutation testing (Additional file 10: Table S3). The significant differential metabolites were identified as OPLS-DA variable importance in the projection (VIP) [32, 33] value > 1 and Student's t-test *P*-value < 0.05, with qualitative names. Their enrichment in KEGG pathways was assessed by Fisher’s exact test.

### 16S rDNA amplicon sequencing of AF mouse model gut microbiota

The fresh fecal samples of the AF mouse model were collected for 16S rDNA amplicon sequencing. Their genomic DNA was extracted, and their V3-V4 variable regions (primer of 16S V3-V4: 341F-806R) were PCR amplified. The target fragments of PCR products were excised and recovered (AxyPrepDNA gel recovery kit, AXYGEN). The PCR products were detected and quantified (QuantiFluor™-ST blue fluorescence quantitative system, Promega). Sequencing library construction was performed by the NEB Next® Ultra™ DNA Library Prep Kit for Illumina (NEB, USA). Operational Taxonomic Units (OTUs) cluster and species annotation sequences analysis were performed by UPARSE-OTU and UPARSE-OTUref algorithms using the UPARSE software package. Sequences with ≥ 97% similarity were assigned to the same OTUs. Taxonomic information was annotated using the Ribosomal Database Project classifier.

Alpha (within a group) and beta (among groups) diversity were calculated by a set of in-house Perl scripts. Calculated alpha diversity index included the diversity index of the flora (Shannon and Simpson), the abundance index of flora (Chao 1 and ACE), the number of OTU (observed species), the sequencing depth index (goods coverage), and phylogenetic diversity index (PD_whole_tree). The beta diversity based on weighted and unweighted unifrac distance was calculated by QIIME software package. The relative abundance of the top 10 phyla among four groups was presented in the Pareto chart.

The non-metric multi-dimensional scaling (NMDS) analysis and ANOSIM test were used to assess the composition differences across the gut microbial communities. The linear discriminant analysis (LDA) effect size (LEfSe) determined the species with a significant impact on the classification of the samples (LEfSe LDA score > 2 and *P-*value < 0.05). Species relative abundance at different taxonomic levels were plotted as a taxonomic cladogram based on LEfSe results.

The PICRUSt software inferred the functional gene composition based on 16S sequencing results. The STAMP differential analysis compared the KEGG and COG functional prediction results (Welch’s t-test analysis) and identified the significantly different functional profiles of gut microbiota between groups.

### Correlation of different metabolites and different gut microbiota

To depict the similarities and differences in the expression patterns of the significantly different metabolites and gut microbiota, a Spearman correlation analysis was used to calculate the correlation coefficients. Then, hierarchical cluster analysis was used to cluster the correlation results. This analysis allows for the identification of clusters of metabolites that show similar correlation patterns with specific gut microbiota.

### Statistics

Continuous variables are expressed as means ± standard deviations or medians (interquartile ranges), and categorical variables are expressed as absolute numbers and percentages. Two groups of continuous variables comparison was conducted by either the student’s t-test (normally distributed data) or the Wilcoxon rank-sum test (non-normally distributed). Three or more groups of continuous variables comparison was conducted by either the ANOVA (normally distributed) or the Kruskal–Wallis test (non-normally distributed). Categorical variables were compared using chi-square statistic tests or Fisher's exact test. Correlation analysis was conducted by Pearson's (parametric data) or Spearman's (non-parametric) analysis. A *P* value of less than 0.05 was considered statistically significant. Statistical analysis was performed on SPSS 20.0. Data drawing was completed by GraphPad Prism 9.0 software and R 4.1.2.

## Results

### Participants baseline characteristics

A total of 60 participants including 30 persistent AF patients and 30 control subjects without AF were enrolled. Table 1 shows the demographic and biochemical characteristics of patients. The ratio of male to female participants was approximately equal. The mean age of participants in the control and AF groups was 57.4 ± 9.9 and 58.3 ± 10.2 years old, respectively. Serum total cholesterol (*P* = 0.008) and low-density lipoprotein cholesterol (*P* = 0.006) levels in AF patients were lower. AF patients exhibited larger left (*P* < 0.001) and right atrial diameter (*P* < 0.001), coupled with lower left ventricular ejection fraction (*P* < 0.001), indicating cardiac function deterioration. No other significant differences in the baseline characteristics were observed.

**Table 1:** Baseline characteristics of persistent atrial fibrillation patients and their control

	Control (n = 30)	Atrial fibrillation (n = 30)	P
Age, y	57.4 ± 9.9	58.3 ± 10.2	0.73
Male/Female	14/16	15/15	1
Body mass index, kg/m^2^	24.09 ± 2.71	25.41 ± 3.52	0.174
Smoking, n (%)	7 (23.3)	5 (16.7)	0.748
Alcohol intake, n (%)	5 (16.7)	5 (16.7)	1
Systolic pressure, mmHg	129.9 ± 16.6	124.7 ± 15.8	0.222
Diastolic pressure, mmHg	82.7 ± 11.8	81.3 ± 14.1	0.675
Hypertension, n (%)	13 (43.3)	17 (56.7)	0.439
Diabetes, n (%)	3 (10.0)	6 (20.0)	0.472
Coronary heart disease, n (%)	7 (23.3)	9 (30)	0.771
Triglyceride (mmol/L)	1.22 (0.95, 2.49)	1.34 (0.96,1.67)	0.229
Total cholesterol (mmol/L)	4.58 ± 0.98	3.97 ± 0.73	0.008
High-density lipoprotein cholesterol (mmol/L)	1.11 ± 0.25	1.15 ± 0.20	0.575
Low-density lipoprotein cholesterol (mmol/L)	2.94 ± 0.82	2.38 ± 0.70	0.006
Free triiodothyronine (pmol/L)	3.25 ± 0.91	3.41 ± 1.62	0.668
Free thyroxine (pmol/L)	4.48 ± 6.01	4.84 ± 6.89	0.839
Thyroid-stimulating hormone (mIU/L)	4.38 ± 8.87	2.54 ± 2.12	0.284
Creatine kinase (U/L)	83.61 ± 36.82	100.99 ± 64.34	0.224
Creatine kinase isoenzyme (CK-MB) (U/L)	17.53 ± 16.81	16.65 ± 5.12	0.798
Alanine aminotransferase (U/L)	19.74 ± 9.57	23.97 ± 14.95	0.204
Aspartate aminotransferase (U/L)	19.80 ± 4.78	22.69 ± 7.01	0.07
Creatinine	84.64 ± 85.31	77.26 ± 17.38	0.644
C-reactive protein	2.07 ± 3.44	1.36 ± 2.35	0.417
Left atrial diameter (mm)	33.07 ± 3.47	43.45 ± 5.08	< 0.001
Right atrial diameter (mm)	31.52 ± 3.21	38.24 ± 5.03	< 0.001
Left ventricular ejection fraction (%)	62.15 ± 3.90	55.86 ± 7.20	< 0.001

### Altered plasma AA profile in persistent AF patients and clinical characteristics

**Fig. 1:**
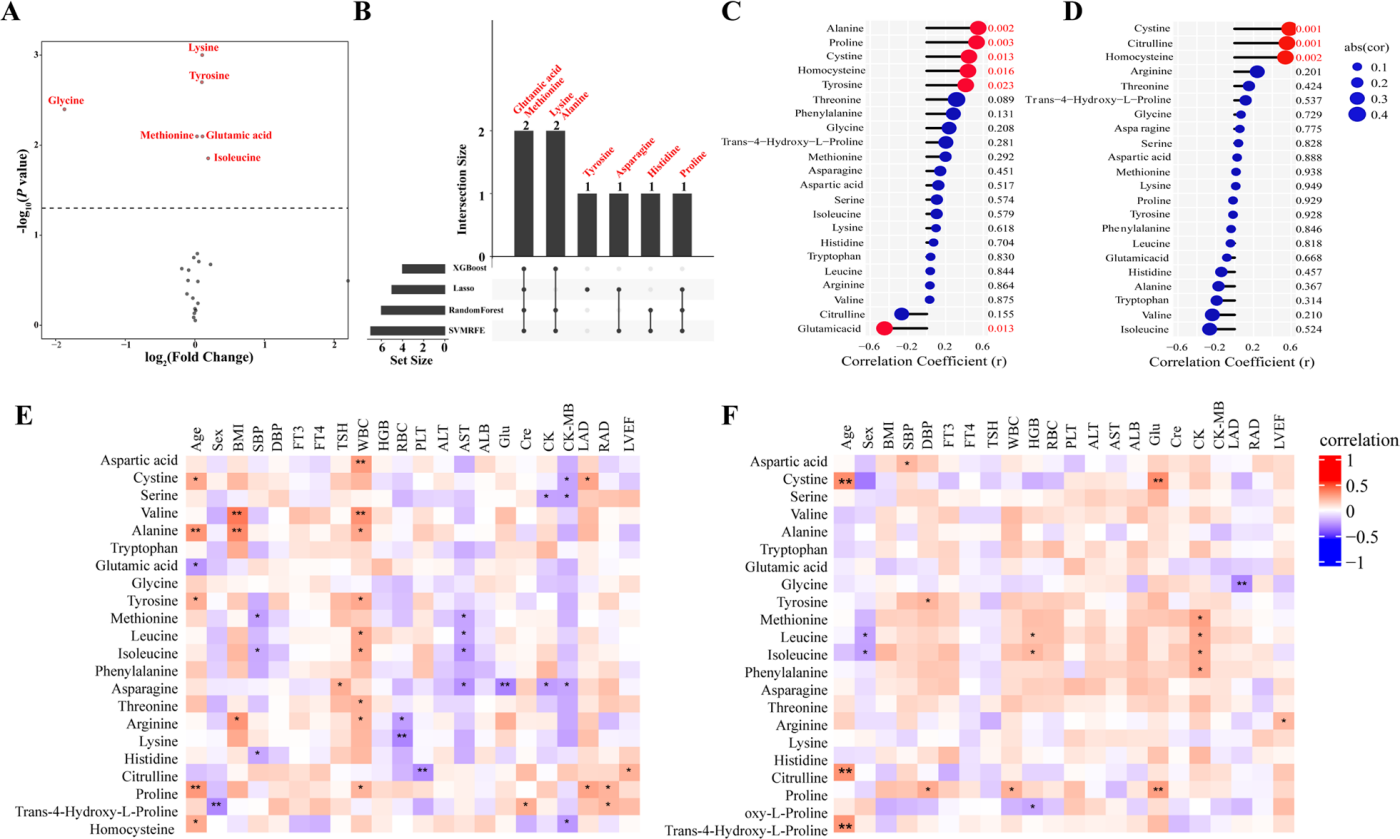
Association of plasma amino acids level and clinical indicators of patients. **A** Volcano plot of the different amino acids between persistent atrial fibrillation patients and control subjects (Student’s t-test, *P* < 0.05). **B** The distribution of feature amino acids screened by each machine learning method. The number of amino acids identified in each subset is represented in the histogram. **C–D** The association of plasma amino acids level and age of **C** control subjects and **D** persistent atrial fibrillation patients. **E**–**F** The association of plasma amino acids level and clinical indicators of **E** control subjects and **F** persistent atrial fibrillation patients. *P < 0.05 and **P < 0.01. BMI, body mass index. SBP, systolic blood pressure. DBP, diastolic blood pressure. FT3, free triiodothyronine. FT4, free thyroxine. TSH, thyroid-stimulating hormone. WBC, white blood cell. HGB, hemoglobin. RBC, red blood cell. PLT, platelets. ALT, alanine aminotransferase. AST, aspartate aminotransferase. ALB, albumin. Glu, blood glucose. BUN, blood urea nitrogen. Cre, creatinine. CK, creatine kinase. CK-MB, creatine kinase isoenzyme. LAD, left atrial diameter. RAD, right atrial diameter. LVEF, Left ventricular ejection fraction

As described in Table 2, a total of 22 plasma AA metabolites were profiled and compared in persistent AF patients and their controls. Higher levels of lysine (Student’s t-test, *P* = 0.001), tyrosine (*P* = 0.002), glutamic acid (*P* = 0.008), methionine (*P* = 0.008), and isoleucine (*P* = 0.014), while a lower level of glycine (*P* = 0.003) were observed in the persistent AF patients (Fig. 1A). Their area under the ROC curve (AUC) ranged from 0.628 to 0.778 (Additional file 1: Figure S1), implying their certain diagnostic efficacy.

**Table 2:** Plasma amino acid profile of persistent atrial fibrillation patients and their control

Amino acid	Control (n = 30)	Atrial fibrillation (n = 30)	P
Alanine	134.2 ± 41.86	124.1 ± 59.3	0.448
Arginine	91.2 ± 50.6	80.1 ± 3.6	0.240
Asparagine	51.4 ± 12.5	48.3 ± 7.0	0.246
Aspartic acid	25.9 ± 3.9	25.4 ± 1.4	0.499
Citrulline	34.5 ± 2.0	34.3 ± 2.1	0.736
Cystine	43.8 ± 1.8	43.3 ± 0.4	0.177
Glutamic acid	25.7 ± 3.2	27.8 ± 2.5	0.008
Glycine	20.9 ± 23.8	5.7 ± 12.0	0.003
Histidine	61.4 ± 4.1	62.9 ± 4.2	0.160
Homocysteine	45.7 ± 0.1	45.8 ± 0.5	0.655
Isoleucine	84.8 ± 17.9	96.8 ± 17.9	0.014
Leucine	50.0 ± 5.5	50.2 ± 4.6	0.883
Lysine	44.3 ± 3.7	47.7 ± 3.6	0.001
Methionine	50.3 ± 1.6	51.4 ± 1.5	0.008
Phenylalanine	65.7 ± 7.0	68.3 ± 8.6	0.196
Proline	112.4 ± 23.1	105.0 ± 33.0	0.318
Serine	8.9 ± 4.5	10.4 ± 4.7	0.211
Threonine	38.3 ± 7.5	37.8 ± 7.2	0.817
Tryptophan	59.1 ± 6.5	60.9 ± 7.6	0.326
Tyrosine	62.8 ± 5.0	67.3 ± 6.0	0.002
Trans-4-Hydroxy-L-Proline	29.6 ± 2.2	29.9 ± 2.6	0.684
Valine	95.8 ± 19.3	98.8 ± 20.8	0.570

The machine learning algorithms, comprising LASSO, SVM-RFE, Random Forest, and XGBoost, identified varying numbers of important AAs: 5 in LASSO screening (glutamic acid, tyrosine, methionine, asparagine, and proline), 7 in SVM-RFE screening (glutamic acid, methionine, lysine, histidine, proline, alanine, and asparagine), 6 in Random Forest (glutamic acid, methionine, lysine, alanine, proline, and histidine), and 4 in XGBoost (glutamic acid, methionine, alanine, and lysine) (Additional file 2: Figure S2). The core intersecting AAs were the glutamic acid and methionine (Fig. 1B).

The alanine, proline, cystine, homocysteine, and tyrosine were positively correlated with age in control group (Fig. 1C). The cystine, citrulline, and homocysteine were positively correlated with age in AF group (Fig. 1D). Notably, while alanine, proline, and tyrosine exhibited positive correlations with age in control group, they exhibited negative correlations with age in AF group. The correlations between AAs and clinical indicators are explored and detailed in Fig. 1E, F.

### Atrial fibrillation mouse model establishment

**Fig. 2:**
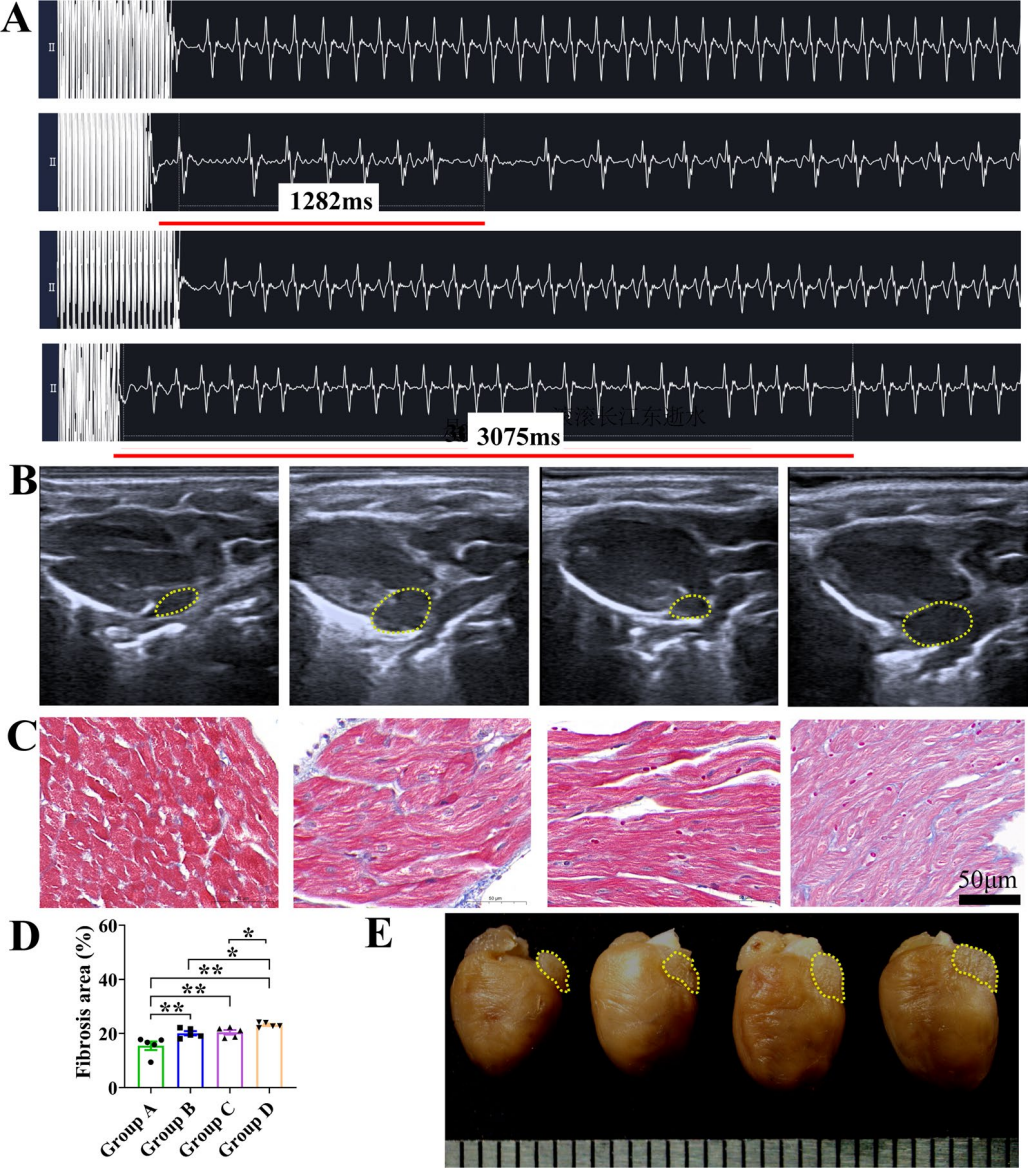
Atrial fibrillation mouse model establishment. **A** Surface electrocardiogram of each group of mice, and the sequence from top to bottom was adult control (Group **A**), adult AF (Group **B**), aged control (Group **C**), and aged AF (Group **D**). The ECG of Group **B** and Group **D** mice showed the atrial fibrillation waveform. **B** Left atrial area detected by M-mode echocardiogram, and the sequence from left to right was Group **A**-**D**. Yellow dashed lines encircle the left atrium. **C**, **D** Representative images of Masson's trichrome staining of the atrial area and measurements of the fibrosis area, and the sequence from left to right was Group **A**–**D** (n = 5 in each group), *P < 0.05; **P < 0.01. **E** Heart gross specimens and the sequence from left to right was Group **A–D**. Yellow dashed lines encircle the left atrium

After four weeks of intravenous injection of Ach and CaCl_2_, the ECG of AF mice (Group B and Group D) showed irregular and rapid AF waveform (Fig. 2A). Atrial fibrillation mice showed more severe myocardial fibrosis than their respective controls and aged AF mice displayed the most severe degree of fibrosis (Fig. 2C, D). Atrial fibrillation mice had larger left atriums shown in both echocardiography and gross specimen, and aged AF mice exhibited the largest left atrium size (Fig. 2B, E). Additionally, aged AF mice exhibited the lowest fractional shortening (FS) and ejection fraction (LVEF) values among all the groups (Additional file 3: Figure S3).

### Altered cardiac metabolites of aged AF mice

**Fig. 3:**
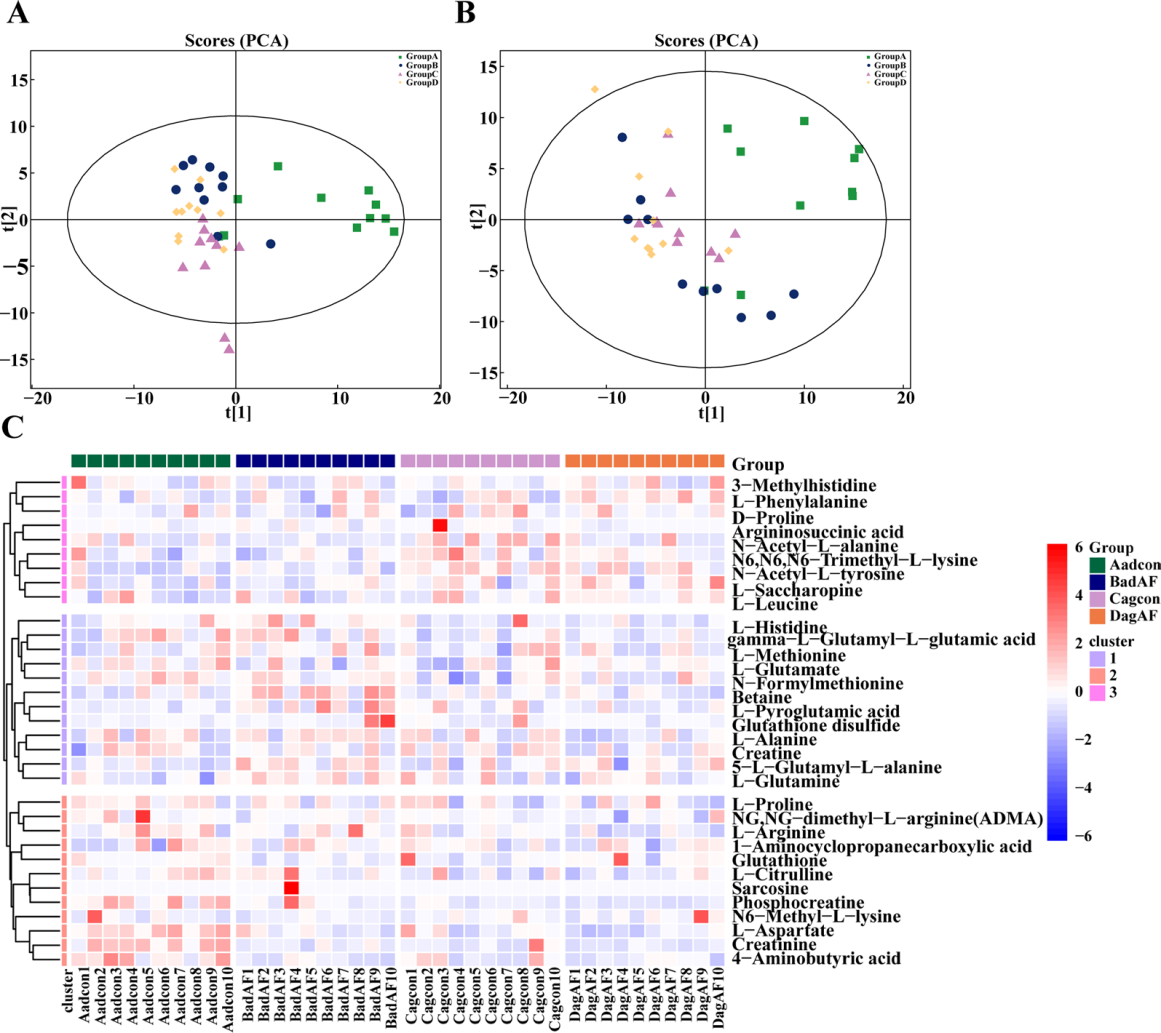
Myocardial untargeted metabolomics and amino acids related metabolites profiles among four mice groups. **A, B** Principal component analysis (PCA) score plots of adult control (Group **A**, Aadcon), adult AF (Group **B**, BadAF), aged control (Group **C**, Cagcon), and aged AF (Group **D**, DagAF) in **A** positive ion mode and **B** negative ion mode. **C** Hierarchical clustering heatmap of amino acids and related metabolites profile of four groups in positive ion mode. n = 10 in each group

Untargeted metabolomics comprehensively identified cardiac metabolic profiles between four mice groups (Additional file 10: Table S1, S2). PCA presented a distinction between them in both positive (POS) and negative (NEG) ion modes (Fig. 3A, B). Significantly different metabolites between groups were defined as OPLS-DA VIP > 1 and *P*-value < 0.05. Adult AF mice compared to adult controls had 48 significantly different metabolites (Additional file 4: Figure S4; Additional file 10: Table S4, S5), including Creatinine, L-Aspartate, Betaine, 4-Aminobutyric acid, Glutathione, L-Pyroglutamic acid, and Phenylacetylglycine. Aged AF mice compared to aged controls had 26 significantly different metabolites (Additional file 5: Figure S5; Additional file 10: Table S6, S7), including L-Phenylalanine. Aged controls compared to adult controls had 43 different metabolites (Additional file 6: Figure S6; Additional file 10: Table S8, 9), including L-Aspartate and Creatinine.

**Fig. 4:**
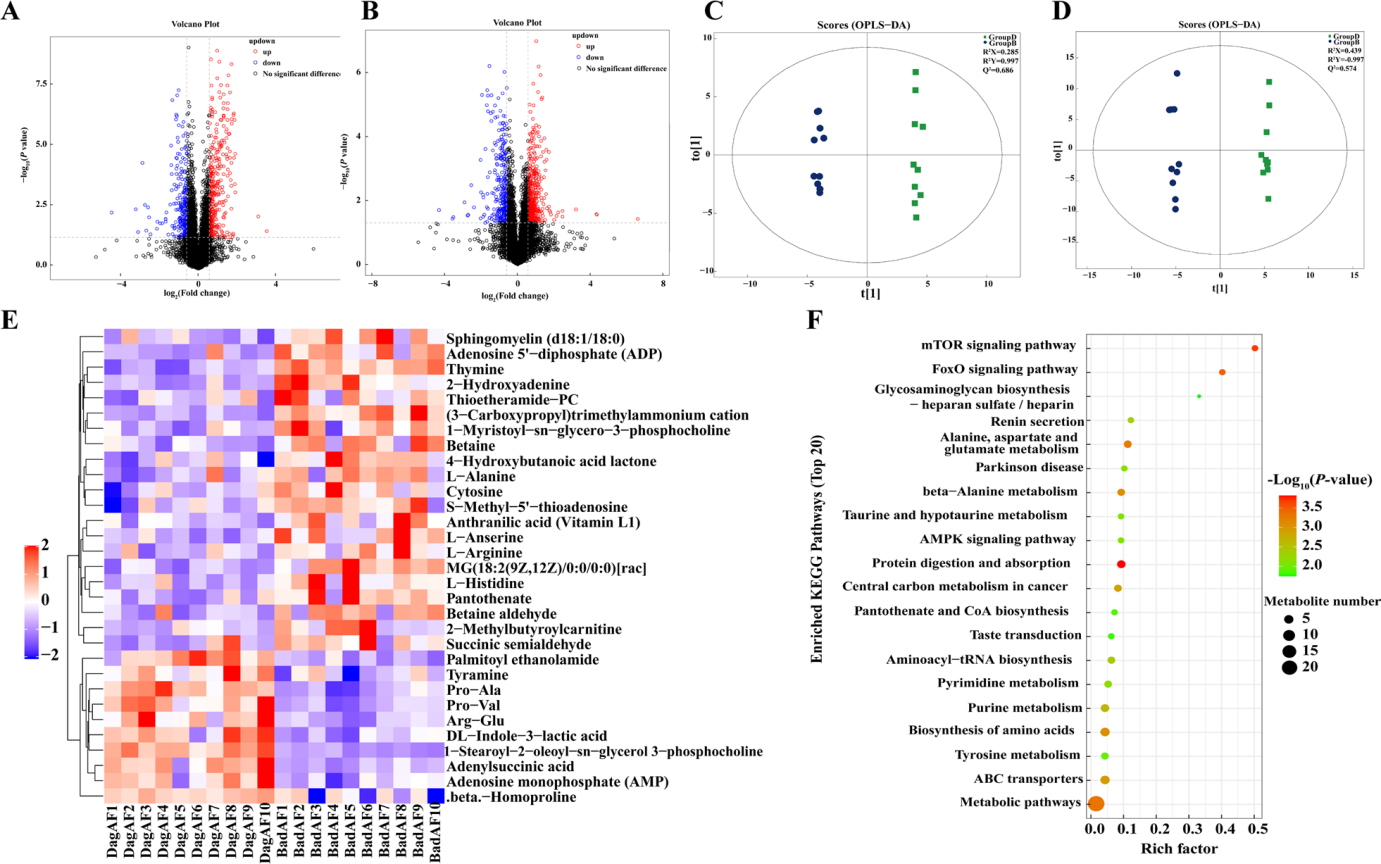
Myocardial untargeted metabolomics between adult AF (Group **B**, BadAF) and aged AF (Group **D**, DagAF) mice. **A**–**B** Volcano plot of the different metabolites in univariate analysis (FC > 1.5 or < 0.67, and t-test* P*-value < 0.05) in **A** positive ion mode and **B** negative ion mode. **C–D** Orthogonal partial least-squares discriminant analysis (OPLS-DA) score plot in **C** positive ion mode and **D** negative ion mode. **E** Hierarchical clustering heatmap of metabolites with significant differences (OPLS-DA VIP value > 1 and t-test *P*-value < 0.05, with qualitative names) in positive ion mode. **F** Top 20 enriched KEGG pathways of the significantly different metabolites in positive ion mode. n = 10 in each group

When comparing aged AF and adult AF mice, differential myocardial metabolites detected in univariate analysis (FC > 1.5 or FC < 0.67 and *P*-value < 0.05) were shown in the volcano plot (Fig. 4A, B). The multi-dimensional statistical analyses OPLS-DA for aged AF vs. adult AF mice in positive ion mode showed strong model statistics for outcome (R^2^Y = 0.997) and reproducibility (Q^2^ = 0.686, 7-fold cross-validation) (Fig. 4C, D). In total, there were 31 and 10 significantly different metabolites in POS and NEG ion mode, respectively (Fig. 4E, Additional file 7: Figure S7; Additional file 10: Table S10, 11). Aged AF mice had significantly lower levels of amino acids, peptides, and analogues, including Betaine (VIP = 8.57, FC = 0.72, *P* = 0.002), L-histidine (VIP = 5.68, FC = 0.51, *P* = 0.008), L-alanine (VIP = 2.81, FC = 0.80, *P* = 0.012), and L-arginine (VIP = 5.41, FC = 0.58, *P* = 0.034). Although the L-Pyroglutamic acid (VIP = 2.49, FC = 0.62, *P* = 0.054) and L-Citrulline (VIP = 1.37, FC = 0.71, *P* = 0.098) were not statistically different in two groups, they were also relatively lower in aged AF mice. These AAs and related metabolites that varied in aged and adult AF mice were the ones associated with age-related AF. The identified differential metabolites enriched in KEGG pathways of alanine, aspartate, glutamate metabolism, beta-alanine metabolism, and tyrosine metabolism (Fig. 4F). Four groups exhibited unique expression profiles of myocardial AAs and related metabolites (Fig. 3C).

### Dysbacteriosis of aged AF mice

**Fig. 5:**
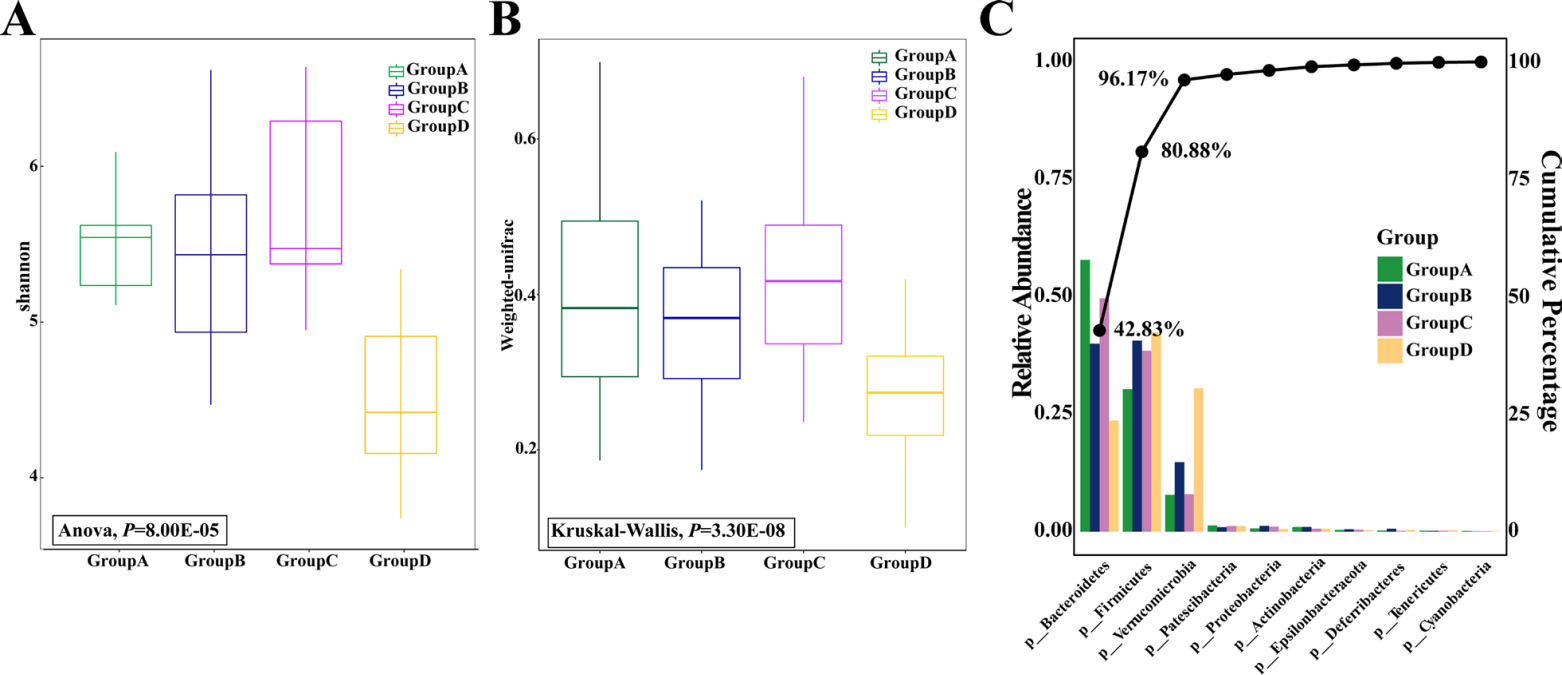
Gut microbiota diversity and relative abundance among four mice groups. **A** Gut microbiota alpha diversity was different among groups based on the Shannon index (*P* = 8.00E-05). **B** Gut microbiota beta diversity was different among groups based on Weighted Unifrac distance (*P* = 3.30E-08). **C** Pareto chart illustrating the relative abundance of the top 10 gut microbiota at the phylum level among four groups. Bars are arranged in ascending order of frequency. The Pareto line indicates cumulative percentage, highlighting that the top 3 gut microbiota contribute to 96.17% of the total abundance. Group **A**: adult control, Group **B**: adult AF, Group **C**: aged control, and Group aged AF, n = 10 in each group

The alpha diversity (Shannon, *P* = 8.00E-05; Simpson, *P* = 5.00E-06; PD_whole_tree, *P* = 1.03E-02) and beta diversity (Weighted unifracbeta distances, *P* = 3.30E-08; Unweighted unifracbeta distances, *P* = 1.18E-05) of mice gut microbiota were significantly different among four groups (Additional file 10: Table S12, S13). The aged AF mice had significantly lower alpha diversity (Fig. 5A) and beta diversity (Fig. 5B) compared with the other groups.

The species abundance clustering at the phylum level revealed distinctive compositions across four groups (Fig. 5C). Based on the cumulative percentage presented in the Pareto line, the top 3 gut microbiota phyla (p__Bacteroidetes, 42.83%; p__Firmicutes, 38.05%; p__Verrucomicrobia, 15.29%) collectively contributed to 96.17% of the total abundance and their relative abundance in each group varies. The relative abundance of Bacteroidetes exhibited significant differences among groups (*P* = 5.76E-07). It was the predominant phylum in control groups, with a relative abundance of 58% in Group A and 50% in Group C. However, in AF groups, Firmicutes dominated as the most prevalent phylum, with 41% in Group B and 43% in Group D. Although Firmicutes did not show significant differences among groups (*P* = 5.73E-02), the Firmicutes/Bacteroidetes ratio (F/B ratio) did (*P* = 1.83E-04). The F/B ratio was higher in AF groups (0.59 in Group A, 0.83 in Group C, 1.23 in Group B, and 2.11 in Group D). Additionally, the relative abundance of Verrucomicrobia exhibited significant differences (*P* = 3.00E-06). AF groups displayed relatively higher levels of Verrucomicrobia compared to control groups (8% in Group A, 8% in Group C, 14% in Group B, and 30% in Group D).

**Fig. 6:**
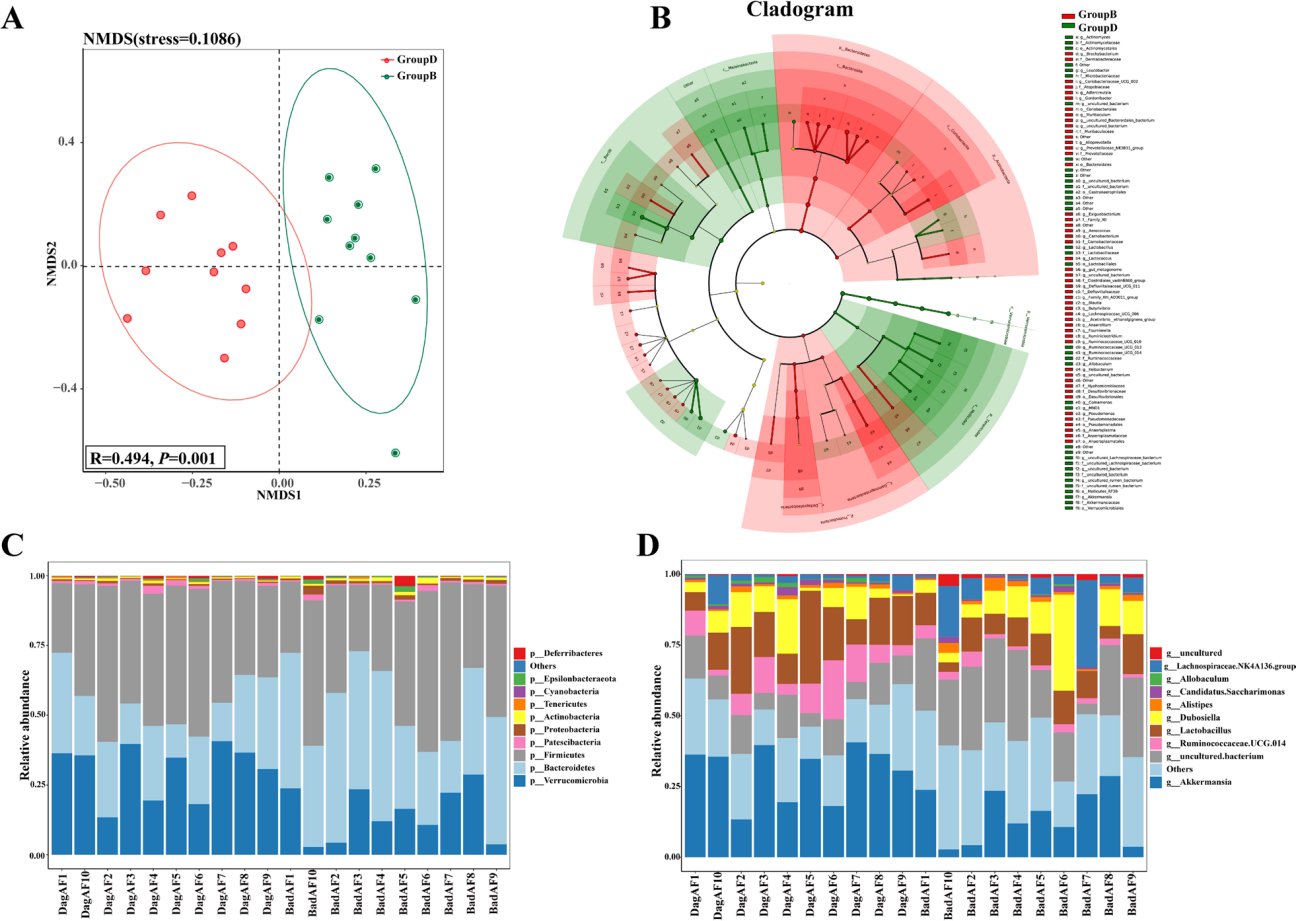
Gut microbiota composition of adult AF (Group **B**, BadAF) and aged AF (Group **D**, DagAF) mice. **A** Non-metric multi-dimensional scaling (NMDS) and ANOSIM test (the box within NMDA plot). **B** The taxonomic cladogram plotted from LEfSe analysis. The red and green nodes represent species with significantly more abundance (LEfSe LDA score > 2 and *P*-value < 0.05) in Group **B** and Group **D**, respectively. **C**-**D** The community composition of the top 10 representative species and their relative abundance at the phylum level (**C**) and the genus level (**D**). n = 10 in each group

Aged AF mice and adult AF mice gut microbiota presented distinct clusters in the non-metric multi-dimensional scaling (NMDS) analysis (Fig. 6A), with significant differences being found in ANOSIM test (R = 0.494, *P* = 0.001). Different gut microbiota with taxonomic levels from phylum to genus were presented in a taxonomic cladogram (LEfSe LDA score > 2 and *P*-value < 0.05), including 5 phyla, 8 classes, 10 orders, 21 families, and 43 genera (Fig. 6B).

**Fig. 7:**
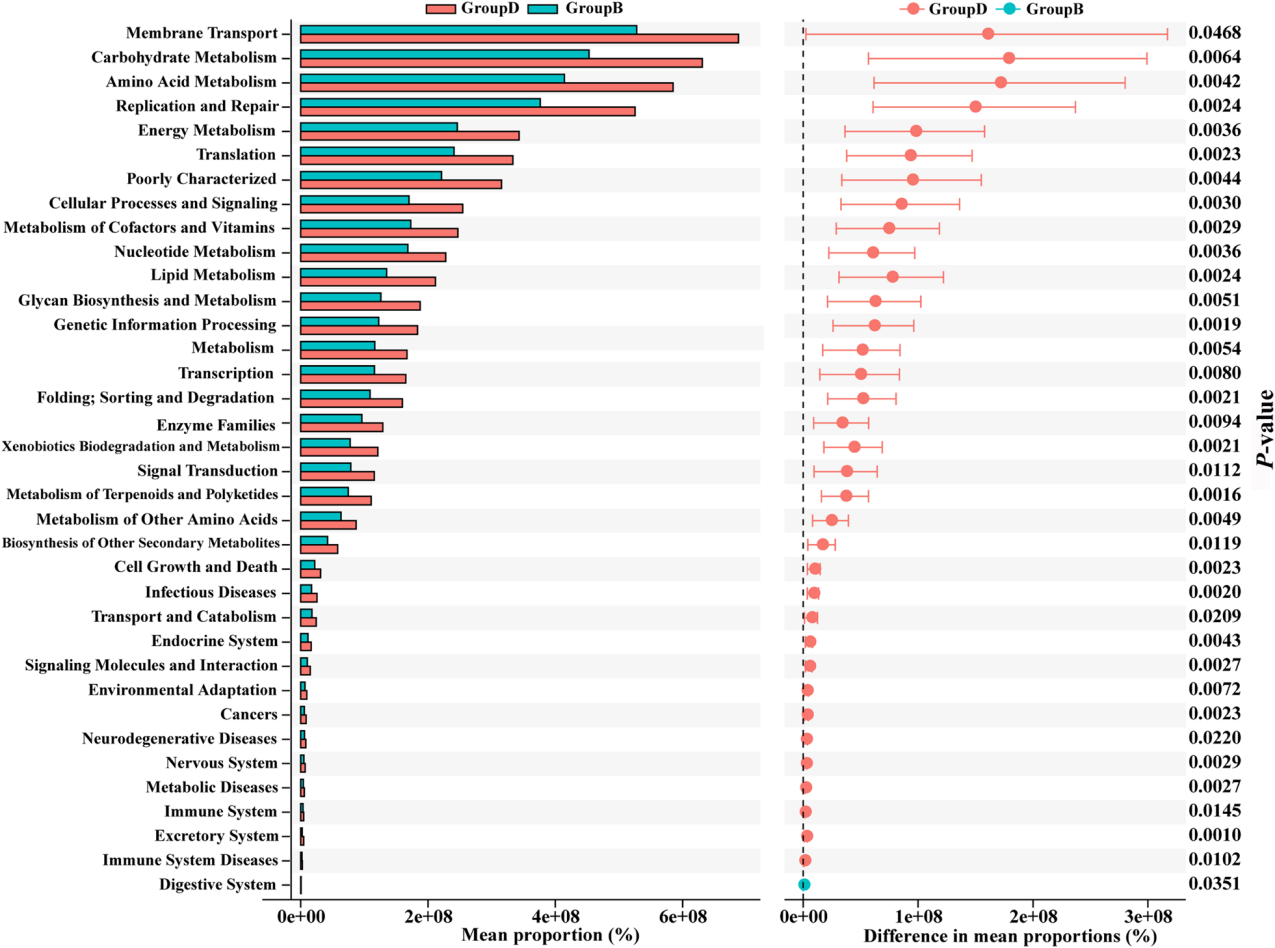
KEGG functional predictive analysis of gut microbiota between adult AF (Group B) and aged AF (Group D) mice conducted by STAMP differential analysis

The relative abundance of the 10 most abundant taxa at the phylum and genus levels is shown in Fig. 6C, D. At phylum level, aged AF mice had significantly more Bacteroidetes (*P* = 1.37E-02), higher F/B ratio (*P* = 4.39E-02), and more Verrucomicrobia (*P* = 1.96E-03). At the genus level, aged AF mice had significantly more Akkermansia (15% in Group B and 31% in Group D, *P* = 1.95E-03). Aged AF mice showed an increased enrichment level of AA metabolism in the KEGG pathway and an increased enrichment level of AA transport and metabolism in the COG pathway (Fig. 7, Additional file 8: Figure S8).

### Correlation between altered metabolites and gut microbiota in aged AF mice

The 41 significantly different metabolites (OPLS-DA VIP > 1 and t-test *P*-value < 0.05) from untargeted metabolomics and 38 significantly different species at the genus level (LEfSe LDA > 2 and *P*-value < 0.05) from 16S rDNA amplicon sequencing analysis were obtained from adult AF and aged AF groups. The correlation between them was displayed as a hierarchical clustering heatmap (Additional file 9: Figure S9).

## Discussion

Given that advanced age is a widely recognized risk factor for AF, healthy aging may impede AF progression. An understanding of mechanisms of age-related AF may provide novel biomarkers and therapeutic targets for AF management.

There is a growing awareness of the detrimental effects of AA disturbances observed in cardiovascular diseases [16]. Our study revealed AA metabolic profile disturbances in age-related AF. Initially, plasma AA variations were detected between persistent AF patients and control subjects, and the correlation between AA levels and age differed. Then, we constructed the AF mouse model and we observed increased myocardial fibrosis, enlarged left atrium, and pronounced cardiac dysfunction in aged AF mice. Besides, we found myocardial AA and related metabolites associated with age-related AF, including Betaine, L-histidine, L-alanine, L-arginine, L-Pyroglutamic acid, and L-Citrulline. They had lower levels in aged AF mice compared with adult AF mice, and it cannot be solely attributed to the aging process, as similar changes were not observed between adult control and aging control mice. Additionally, aged AF mice had less diverse gut microbiota, more severe gut dysbacteriosis, and predicted gut microbiota AA metabolic disturbance.

Not only did AF patients have elevated plasma glutamic acid levels, but AF mice also had elevated myocardial glutamic acid derivative, the pyroglutamic acid. Given that glutamic acid is one of the most abundant amino acids in the body, its metabolic versatility has sparked emerging interest in its role in cardiovascular diseases [34–36]. One of its crucial molecular mechanisms is the maintenance of glutathione levels. Glutathione, synthesized from glutamate, cysteine, and glycine, undergoes hydrolysis to yield pyroglutamic acid [37]. It was reported that AF patients had significantly lower left atrial glutathione levels, linked to decreased atrial contractility and reduced L-type calcium current [38] in atrial myocytes [39]. The observed lower glycine levels in AF patients and lower glutathione in AF mice in our study suggested a potential disruption of the glutathione cycle during AF.

The myocardial levels of alanine and its associated metabolic pathways differed between aged AF and adult AF mice. Aged AF mice had lower alanine levels. Intriguingly, a negative correlation between alanine levels and age was observed in AF patients. Alanine supplementation has been documented to improve fatigue, increase strength, and enhance cognitive function in the elderly [40, 41]. These health benefits of alanine for the elderly suggest its plausible benefits in age-related AF.

Arginine (Arg) is oxidized to nitric oxide (NO) and L-citrulline (Cit) by nitric oxide synthase (NOS). The implications of the NOS-NO pathway in cardiovascular diseases have been extensively studied [42, 43]. Diminished levels of Arg, which serves as a precursor of NO, have been associated with an increased risk of major adverse cardiovascular events [44]. The observed reduction of Arg and Cit in our study implied defective arginine metabolism and reduced availability of NO in aged AF. Moreover, Arg administration and co-administration of Arg and Cit enhanced NO availability and rectified diverse metabolic disorders [45–47]. Our findings suggested that combined supplementation of Arg and Cit may be beneficial for age-related AF.

Previous genomic and physiological studies demonstrated that gut microbiota has specialized enzymes that utilize AAs [48]. Gut microbial proteolysis undigested protein and peptides and produced various microbial metabolites [49]. Some of these bacterial metabolites are then transported to portal blood, where they exert diverse physiological effects on the circulation and peripheral organs [50]. Metabolic disorders are associated with gut microbiota composition and function, and microbiota-derived metabolites, including bile acids, short-chain fatty acids, BCAAs, tryptophan, and so on [51, 52]. Previous studies observed that disturbed gut microbiota was associated with the onset of AF [53], the type of AF [54], and the duration of AF [55]. Our finding not only found the dysbacteriosis in age-related AF but also suggested a disruption of gut microbiota AA metabolism.

Our studies have certain limitations. Firstly, the observed AA disturbances in age-related AF can only suggest a correlation, but not causality. Their correlation suggested AA disturbances may serve as possible biomarkers of AF, and restoring AA homeostasis as an AF intervention needs to be verified in subsequent research.

Secondly, the absolute numerical differences of AAs between AF patients and counterparts were not large and their AUC value prompts moderate diagnostic efficacy. This observation may be attributed to the relatively limited number and the heightened metabolic heterogeneity of enrolled patients. Thus, further research within larger cohorts stratified by age, gender, AF duration, and metabolic status is still expected to deliver more clear results.

Thirdly, plasma samples may not capture altered AA metabolites in the atrial tissue of AF patients. We analyzed myocardial tissue from AF mouse model instead. Notably, the inherent complexity of metabolomic data, along with variations between different organisms and tissues, limits us to identifying a large number of consistent metabolic signatures. However, a definitive conclusion can be drawn: substantial disturbances of AA metabolism exist in both AF patients and AF mice.

Despite these limitations, our research also possesses several strengths. Firstly, we provide a comprehensive network of AA disturbance in age-related AF from multiple dimensions, including plasma, myocardium, and gut microbiota. Secondly, we found plasma AA disturbance in AF patients. Being easily detectable, plasma AAs may serve as highly feasible AF biomarkers. Thirdly, we constructed the AF mouse model and identified various AA disturbances related to AF. The health benefits of restoring these AAs have been proved in previous studies. Considering the involvement of AAs in aging and cardiovascular diseases, it is worth contemplating the potential benefits of restoring AA homeostasis in age-related AF.

## Conclusions

This study provided a comprehensive network of AA disturbances in age-related AF from multiple dimensions, including plasma, myocardium, and gut microbiota. Disturbances of AAs may serve as AF biomarkers, and restoring their homeostasis may have potential benefits for the management of age-related AF.

### Supplementary Information

**Additional file 1: Figure S1.** Receiver operating characteristic (ROC) curve of altered plasma amino acids. The area under the ROC curve (AUC) and* P*-value for each amino acid were shown.

**Additional file 2: Figure S2.** Feature amino acids identification by machine learning methods. (A-B) LASSO screening identified 5 feature amino acids (glutamic acid, tyrosine, methionine, asparagine, and proline). (C) SVM-RFE screening identified 7 feature amino acids (glutamic acid, methionine, lysine, histidine, proline, alanine, and asparagine). (D-E) Random Forest model identified 6 feature amino acids sorted by mean decrease accuracy and mean decrease Gini index (glutamic acid, methionine, lysine, alanine, proline, and histidine). (F) 10-fold cross-validation of XGBoost to determine the optimal number of rounds. (G) XGBoost algorithm identified 4 important amino acids (glutamic acid, methionine, alanine, and lysine). LASSO, least absolute shrinkage and selection operator. SVM-RFE, Support Vector Machine-Recursive Feature Elimination. XGBoost, Extreme Gradient Boosting.

**Additional file 3: Figure S3.** Mice cardiac function evaluated by transthoracic M‐mode echocardiogram. (A-D) The M‐mode echocardiography of Group A (A), Group B (B), Group C (C), and Group D (D). (E-H) The M‐mode echocardiography parameters LVIDd, LVIDs, FS (%), and LVEF (%). n = 10 in each group. *P < 0.05; **P < 0.01; ***P < 0.001. LVIDd, left ventricular internal dimension in diastole. LVIDs, left ventricular internal dimension in systole. FS, fractional shortening. LVEF, Left ventricular ejection fraction.

**Additional file 4: Figure S4.** Hierarchical clustering heatmap of myocardium metabolites with significant differences between adult control (Group A, Aadcon) and adult AF (Group B, BadAF) mice. (A) in positive ion mode. (B) in negative ion mode. n = 10 in each group

**Additional file 5: Figure S5.** Hierarchical clustering heatmap of metabolites with significant differences between aged control (Group C, Cagcon) and aged AF (Group D) mice. (A) in positive ion mode. (B) in negative ion mode. n = 10 in each group

**Additional file 6: Figure S6.** Hierarchical clustering heatmap of metabolites with significant differences between adult control (Group A, Aadcon) and aged control (Group C, Cagcon) mice. (A) in positive ion mode. (B) in negative ion mode. n = 10 in each group

**Additional file 7: Figure S7.** Hierarchical clustering heatmap of metabolites with significant differences between adult AF (Group B, BadAF) and aged AF (Group D, DagAF) mice in negative ion mode

**Additional file 8: Figure S8.** COG functional predictive analysis of gut microbiota between adult AF (Group B) and aged AF (Group D) mice conducted by STAMP differential analysis

**Additional file 9: Figure S9.** Correlation analysis between significantly different gut microbial genera and significantly different myocardial metabolites in adult AF (Group B) and aged AF (Group D) mice. In a hierarchical clustering heatmap, each row represents a significantly different gut microbial genera (LEfSe LDA > 2 and *P*-value < 0.05) from 16S rDNA amplicon sequencing analysis, and each column represents a significantly different metabolite (OPLS-DA VIP > 1 and t-test *P*-value < 0.05) from untargeted metabolomics. Positive correlations (correlation coefficient *r* > 0) are depicted in red, while negative correlations (*r* < 0) are depicted in blue. The *P*-value reflects the level of significance of the correlation. *P < 0.05, **P < 0.01 and ***P < 0.001.

**Additional file 10.** Supplementary tables.

## Data Availability

All data are included in the manuscript or in the Supplementary Materials and are available from the corresponding authors upon request with publication of this manuscript. Source data are provided with this paper.
