# Supplementary figures and images for: Amino acid profile alteration in age-related atrial fibrillation
Source: J Transl Med. 2024 Mar 9;22:259. doi: 10.1186/s12967-024-05028-7 (PMC10925006; doi:10.1186/s12967-024-05028-7)

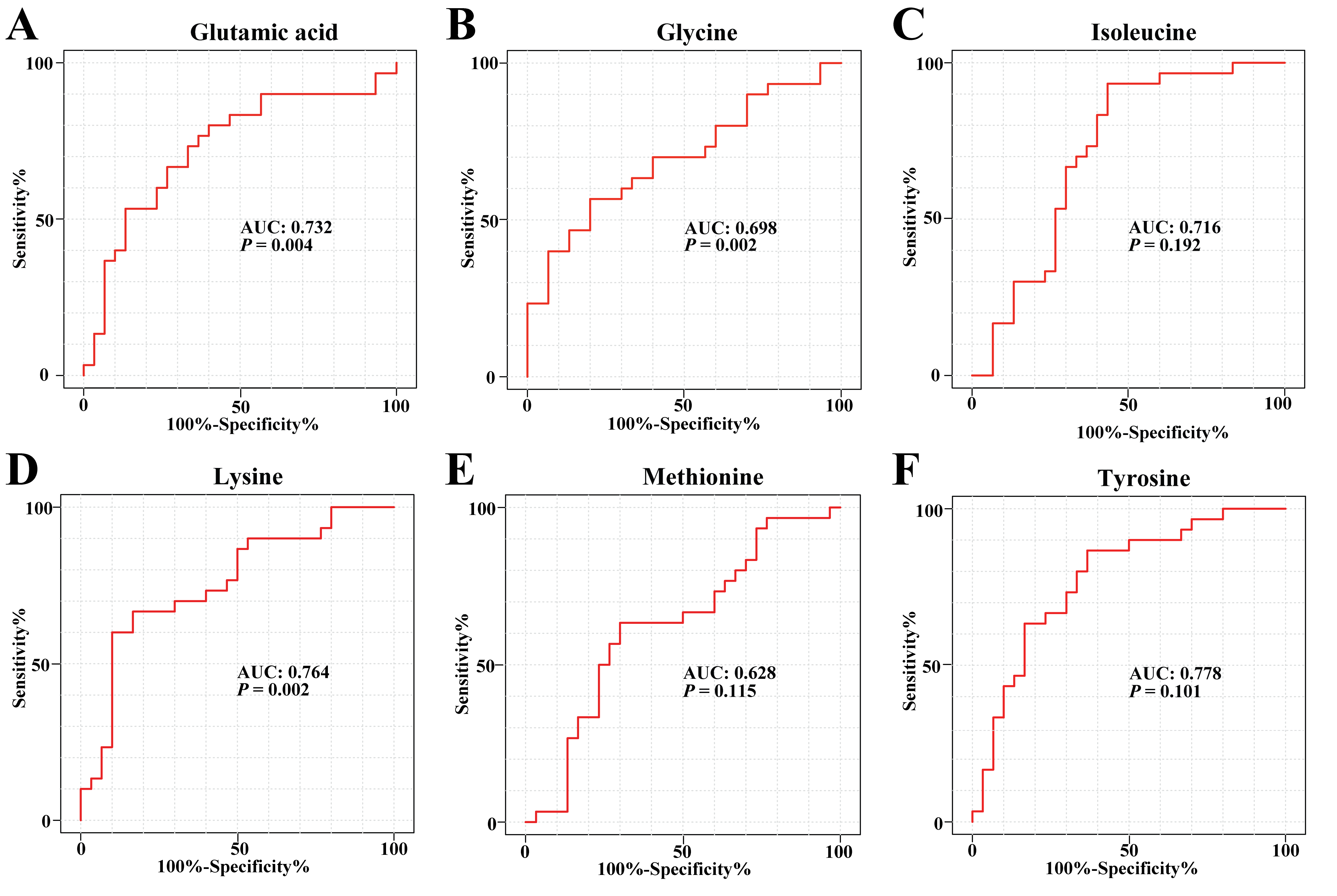

Supplement: Supplementary file 1 — Additional file 1: Figure S1. Receiver operating characteristic (ROC) curve of altered plasma amino acids. The area under the ROC curve (AUC) and P-value for each amino acid were shown. [file 12967_2024_5028_MOESM1_ESM.tif]

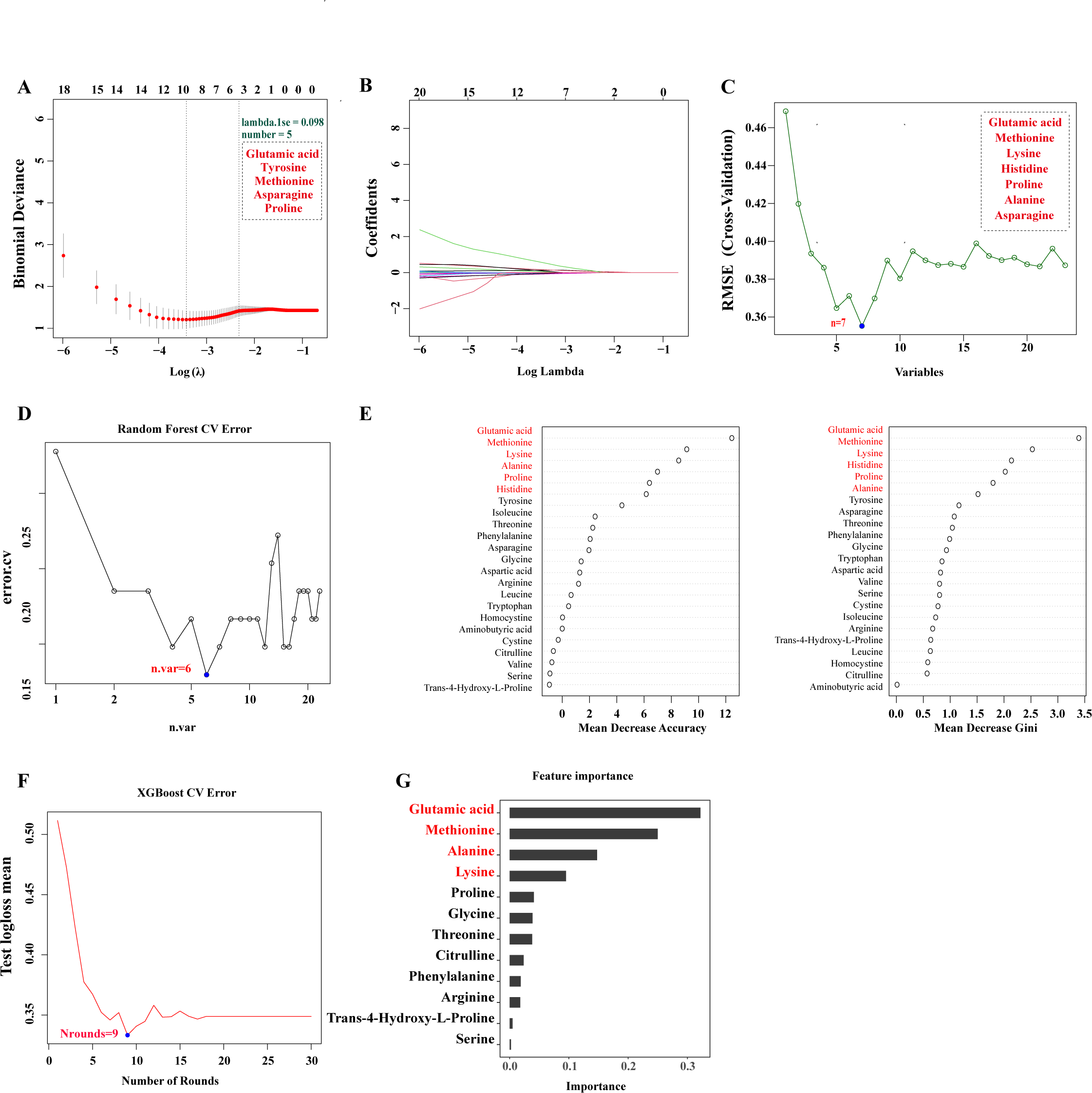

Supplement: Supplementary file 2 — Additional file 2: Figure S2. Feature amino acids identification by machine learning methods. (A-B) LASSO screening identified 5 feature amino acids (glutamic acid, tyrosine, methionine, asparagine, and proline). (C) SVM-RFE screening identified 7 feature amino acids (glutamic acid, methionine, lysine, histidine, proline, alanine, and asparagine). (D-E) Random Forest model identified 6 feature amino acids sorted by mean decrease accuracy and mean decrease Gini index (glutamic acid, methionine, lysine, alanine, proline, and histidine). (F) 10-fold cross-validation of XGBoost to determine the optimal number of rounds. (G) XGBoost algorithm identified 4 important amino acids (glutamic acid, methionine, alanine, and lysine). LASSO, least absolute shrinkage and selection operator. SVM-RFE, Support Vector Machine-Recursive Feature Elimination. XGBoost, Extreme Gradient Boosting. [file 12967_2024_5028_MOESM2_ESM.tif]

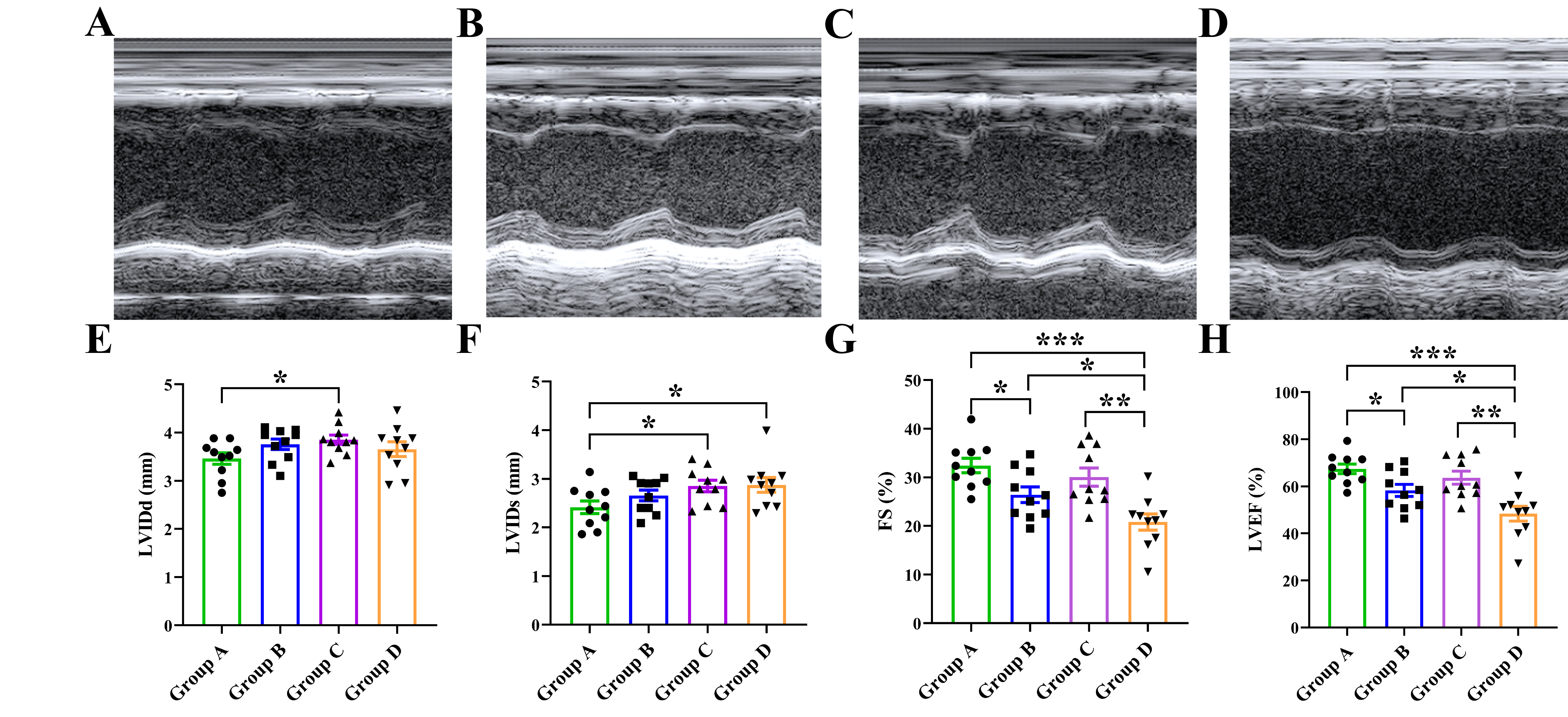

Supplement: Supplementary file 3 — Additional file 3: Figure S3. Mice cardiac function evaluated by transthoracic M‐mode echocardiogram. (A-D) The M‐mode echocardiography of Group A (A), Group B (B), Group C (C), and Group D (D). (E-H) The M‐mode echocardiography parameters LVIDd, LVIDs, FS (%), and LVEF (%). n = 10 in each group. *P < 0.05; **P < 0.01; ***P < 0.001. LVIDd, left ventricular internal dimension in diastole. LVIDs, left ventricular internal dimension in systole. FS, fractional shortening. LVEF, Left ventricular ejection fraction. [file 12967_2024_5028_MOESM3_ESM.tif]

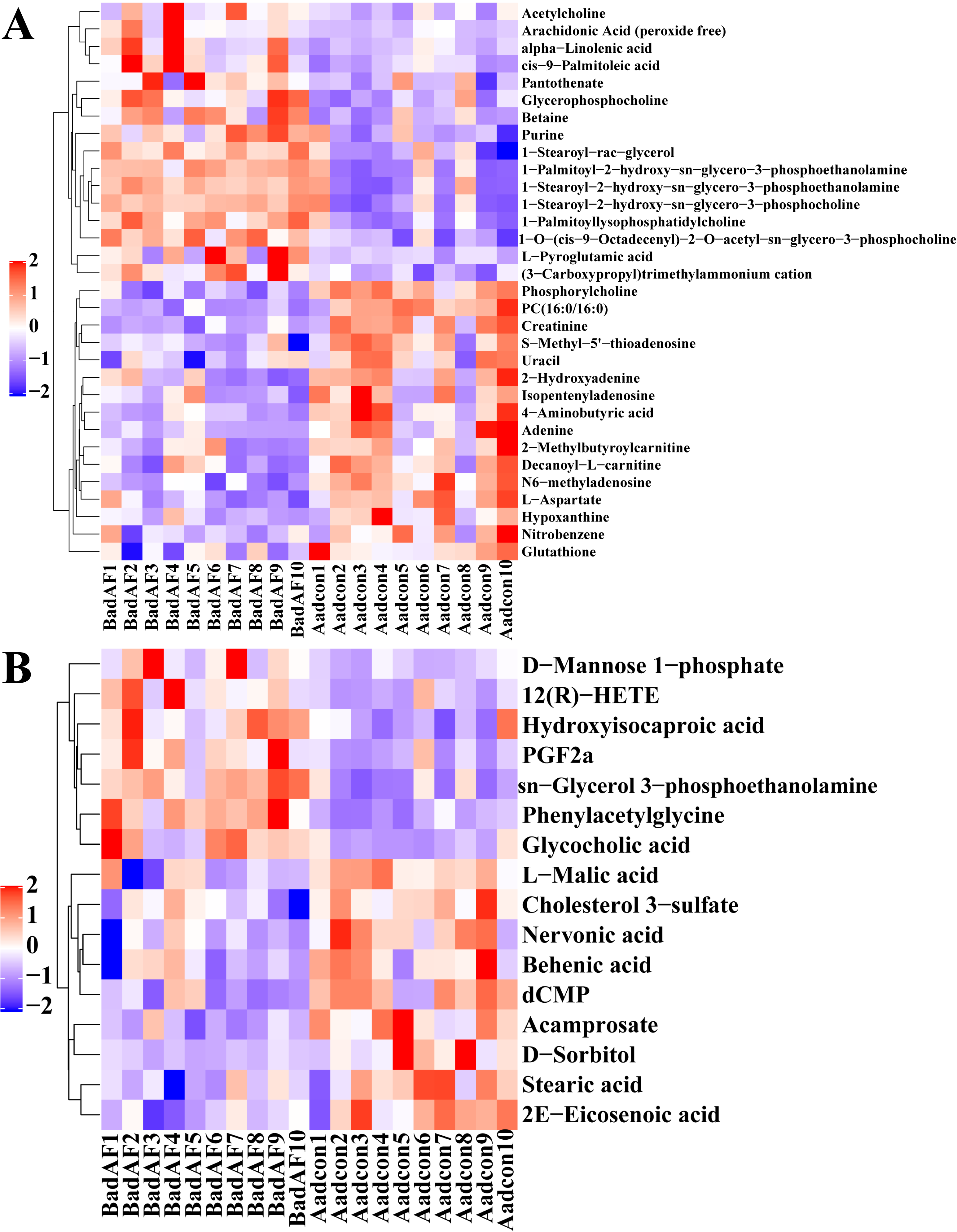

Supplement: Supplementary file 4 — Additional file 4: Figure S4. Hierarchical clustering heatmap of myocardium metabolites with significant differences between adult control (Group A, Aadcon) and adult AF (Group B, BadAF) mice. (A) in positive ion mode. (B) in negative ion mode. n = 10 in each group [file 12967_2024_5028_MOESM4_ESM.tif]

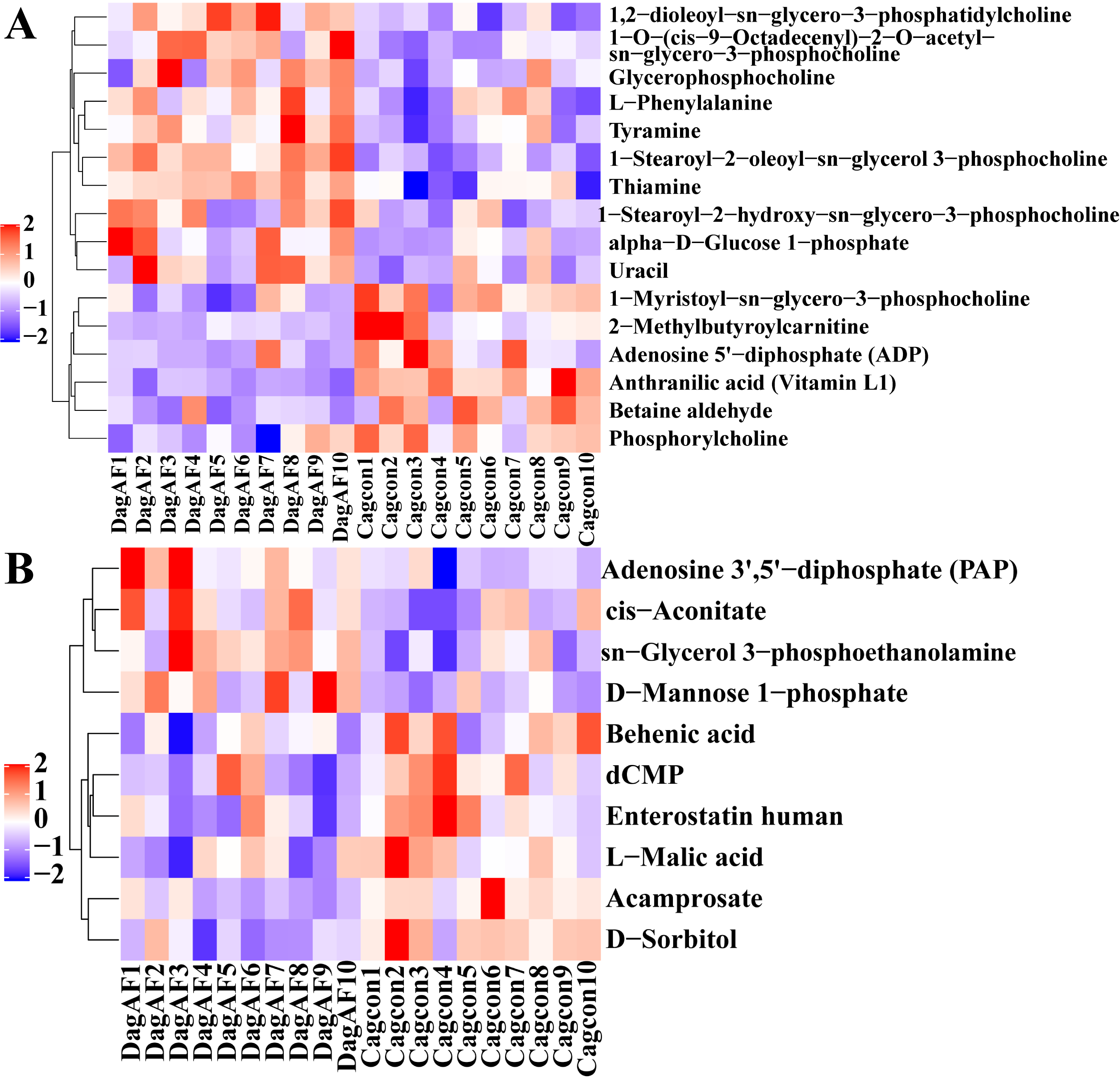

Supplement: Supplementary file 5 — Additional file 5: Figure S5. Hierarchical clustering heatmap of metabolites with significant differences between aged control (Group C, Cagcon) and aged AF (Group D) mice. (A) in positive ion mode. (B) in negative ion mode. n = 10 in each group [file 12967_2024_5028_MOESM5_ESM.tif]

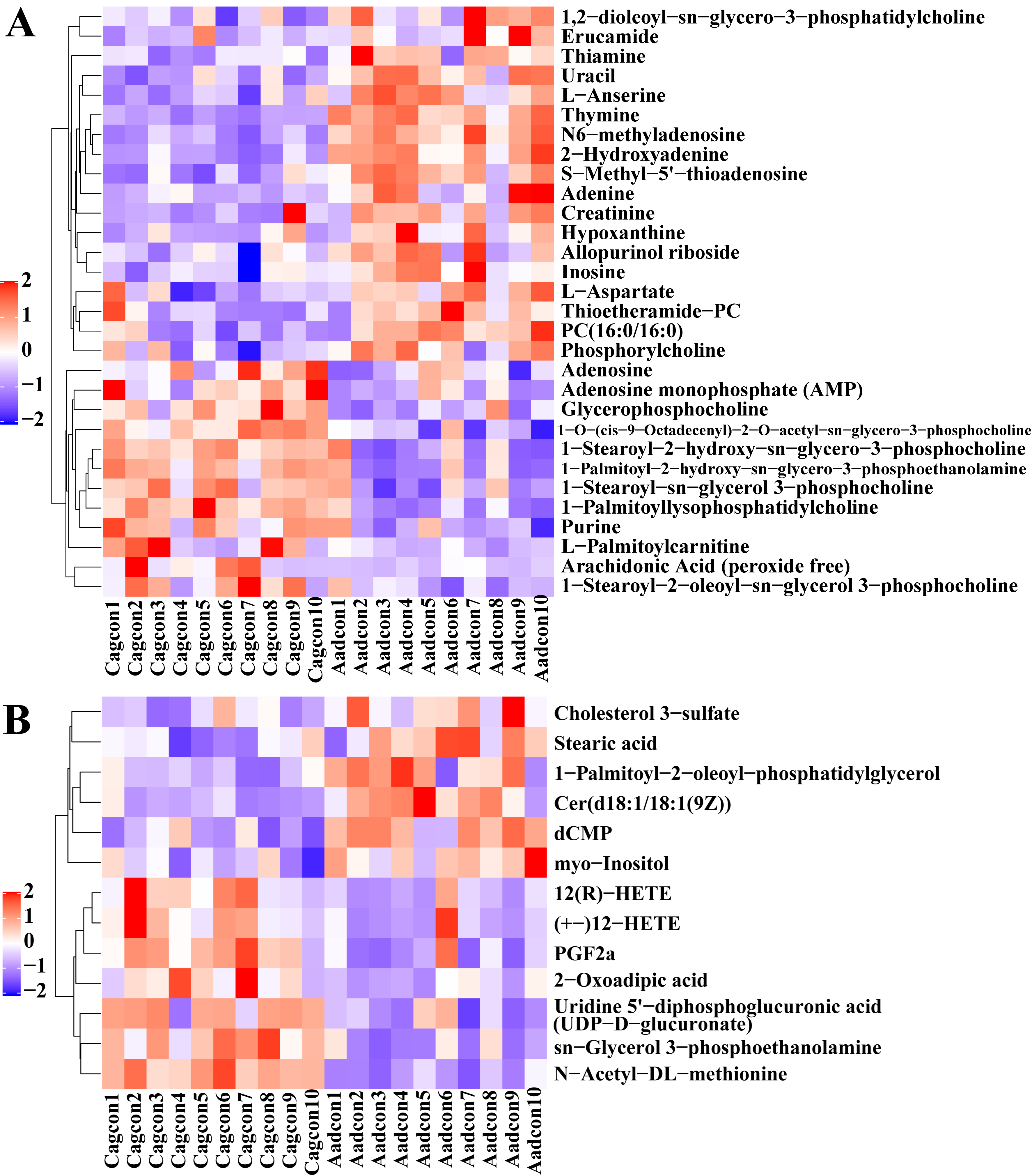

Supplement: Supplementary file 6 — Additional file 6: Figure S6. Hierarchical clustering heatmap of metabolites with significant differences between adult control (Group A, Aadcon) and aged control (Group C, Cagcon) mice. (A) in positive ion mode. (B) in negative ion mode. n = 10 in each group [file 12967_2024_5028_MOESM6_ESM.tif]

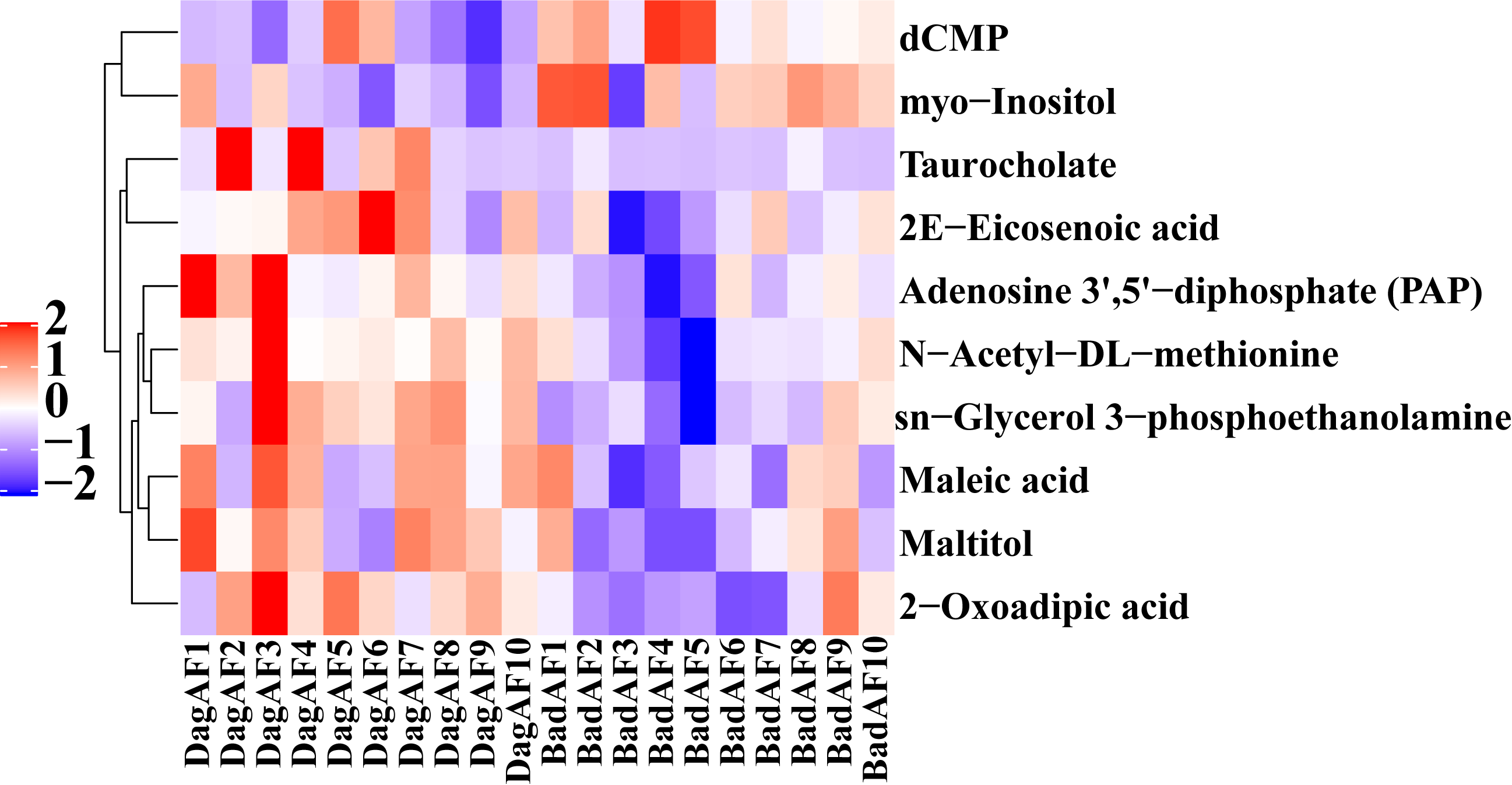

Supplement: Supplementary file 7 — Additional file 7: Figure S7. Hierarchical clustering heatmap of metabolites with significant differences between adult AF (Group B, BadAF) and aged AF (Group D, DagAF) mice in negative ion mode [file 12967_2024_5028_MOESM7_ESM.tif]

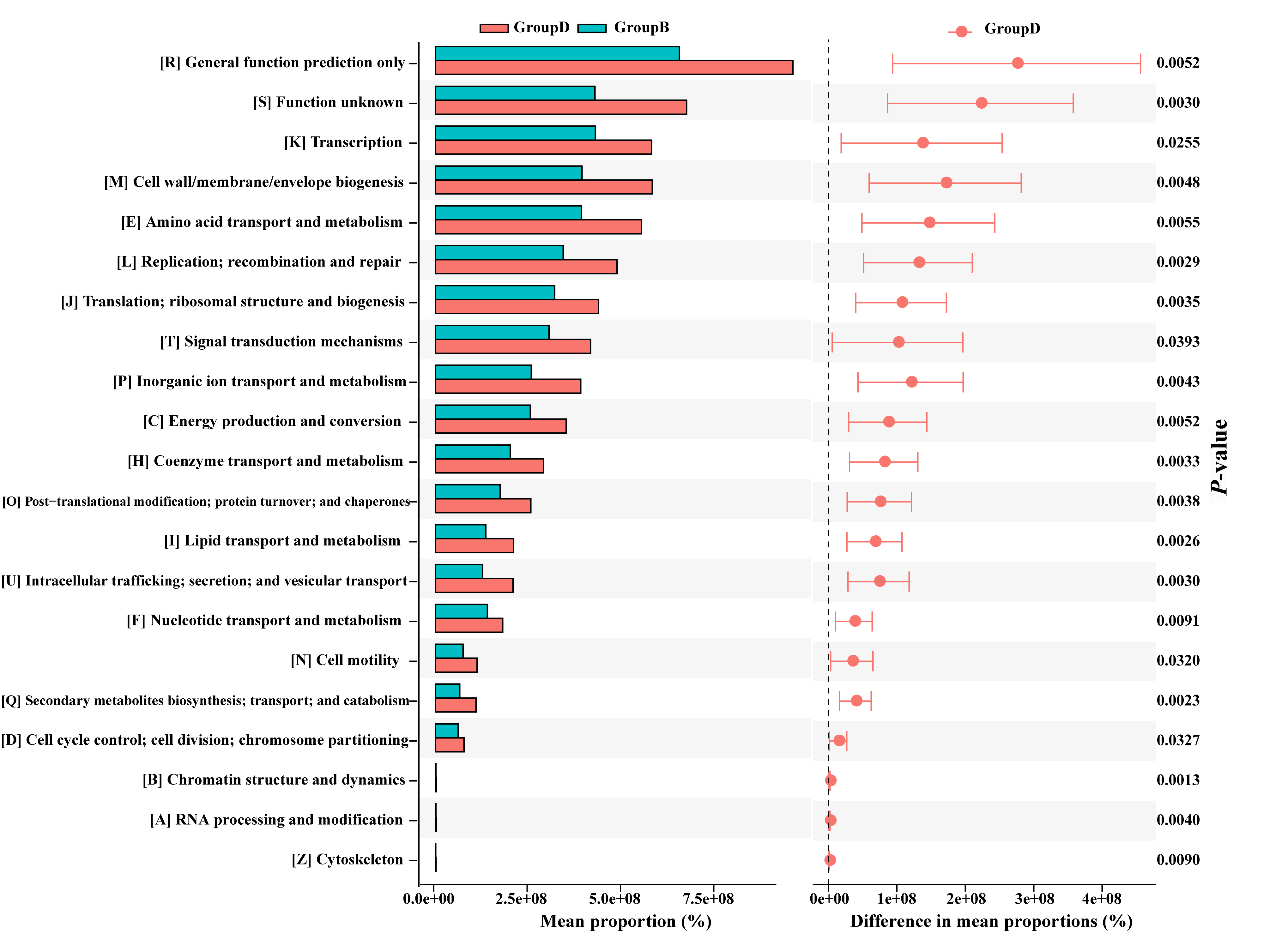

Supplement: Supplementary file 8 — Additional file 8: Figure S8. COG functional predictive analysis of gut microbiota between adult AF (Group B) and aged AF (Group D) mice conducted by STAMP differential analysis [file 12967_2024_5028_MOESM8_ESM.tif]

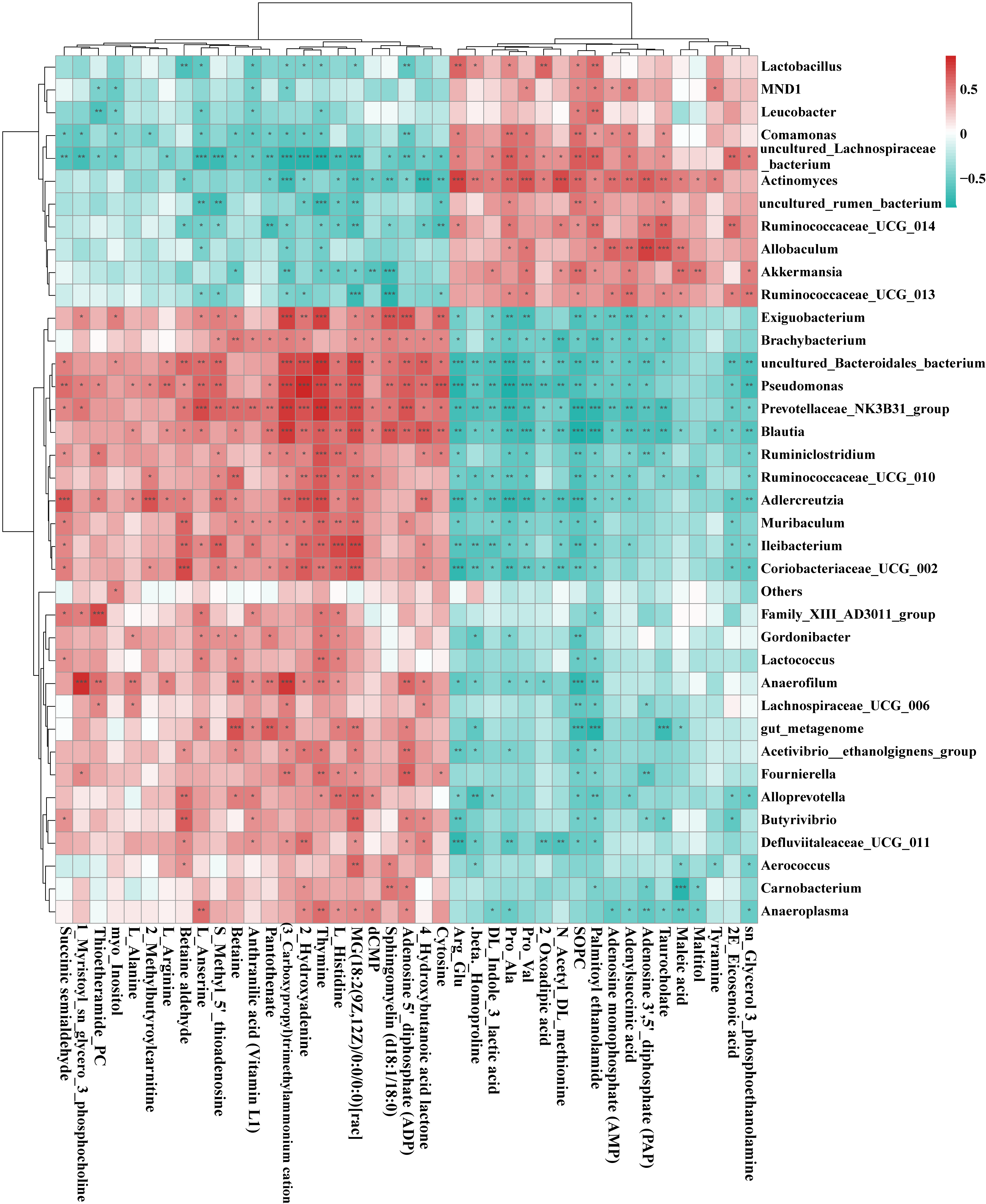

Supplement: Supplementary file 9 — Additional file 9: Figure S9. Correlation analysis between significantly different gut microbial genera and significantly different myocardial metabolites in adult AF (Group B) and aged AF (Group D) mice. In a hierarchical clustering heatmap, each row represents a significantly different gut microbial genera (LEfSe LDA > 2 and P-value < 0.05) from 16S rDNA amplicon sequencing analysis, and each column represents a significantly different metabolite (OPLS-DA VIP > 1 and t-test P-value < 0.05) from untargeted metabolomics. Positive correlations (correlation coefficient r > 0) are depicted in red, while negative correlations (r < 0) are depicted in blue. The P-value reflects the level of significance of the correlation. *P < 0.05, **P < 0.01 and ***P < 0.001. [file 12967_2024_5028_MOESM9_ESM.tif]
